# Efficacy of Probiotics in Rheumatoid Arthritis and Spondyloarthritis: A Systematic Review and Meta-Analysis of Randomized Controlled Trials

**DOI:** 10.3390/nu14020354

**Published:** 2022-01-14

**Authors:** Pauline Sanchez, Jean-Guillaume Letarouilly, Yann Nguyen, Johanna Sigaux, Thomas Barnetche, Sébastien Czernichow, René-Marc Flipo, Jérémie Sellam, Claire Daïen

**Affiliations:** 1 Department of Rheumatology, CHU de Montpellier, Montpellier University, F-34295 Montpellier, France; p-sanchez@chu-montpellier.fr; 2 Department of Rheumatology, CHU Lille, Université de Lille, F-59000 Lille, France; jeanguillaume.letarouilly@chru-lille.fr (J.-G.L.); renemarc.flipo@chru-lille.fr (R.-M.F.); 3 Department of Internal Medicine, Hôpital Beaujon, AP-HP Nord, Université de Paris, F-92100 Clichy, France; yann.nguyen2@aphp.fr; 4 Department of Rheumatology, Hôpital Avicenne, AP-HP, INSERM U1125, Université Paris 13, F-93017 Bobigny, France; johanna.sigaux@aphp.fr; 5 Department of Rheumatology, FHU ACRONIM, Bordeaux University Hospital, F-33076 Bordeaux, France; thomas.barnetche@chu-bordeaux.fr; 6 Department of Nutrition, Specialized Obesity Center, Hôpital Européen Georges Pompidou, Université de Paris, AP-HP, F-75015 Paris, France; sebastien.czernichow@aphp.fr; 7 Epidemiology and Biostatistics Sorbonne Paris City Center, UMR1153, Institut National de la Santé et de la Recherche Médicale, F-75004 Paris, France; 8 Department of Rheumatology, Hôpital Saint Antoine, AP-HP, DMU 3ID, CRSA Inserm UMRS_938, Sorbonne Université, F-75012 Paris, France; jeremie.sellam@aphp.fr; 9 PhyMedExp, University of Montpellier, INSERM U1046, CNRS UMR 9214, F-34295 Montpellier, France

**Keywords:** rheumatoid arthritis, spondyloarthritis, probiotics, microbiota, disease activity

## Abstract

Background: We aimed to provide a systematic review and meta-analysis of randomized controlled trials assessing the effect of probiotics supplementation on symptoms and disease activity in patients with chronic inflammatory rheumatic diseases (rheumatoid arthritis (RA), spondylarthritis (SpA), or psoriatic arthritis). Methods: A systematic literature review and meta-analysis from RA and SpA randomized controlled trials were conducted searching for articles in MEDLINE/PubMed and abstracts from recent international rheumatology meetings. The control group was a placebo or another dietary intervention. The risk of bias of the selected studies was evaluated using the Cochrane Collaboration tool and the Jadad scale. Results: The initial search yielded 173 articles. Of these, 13 studies were included in the qualitative synthesis, 8 concerning a total of 344 RA patients and 2 concerning a total of 197 SpA patients. Three meta-analyses were also analyzed. Probiotic strains and quantities used were different among trials (5 studies using *Lactobacillus* sp., 1 trial *Bacillus* *coagulans* and the others a mix of different probiotic strains). Time to assess response ranged from 8 weeks to one year. Two studies associated probiotic supplementation with a dietary intervention. Meta-analysis showed a statistically significant decrease of C-reactive protein (CRP) concentration (mean difference (MD)) −3.04 (95% CI −4.47, −1.62) mg/L, *p* < 0.001; I^2^ = 20%, *n* patients = 209) with probiotics in RA. However, after excluding high-risk-of-bias trials of meta-analysis, there was no difference between probiotics and placebo on DAS28 (standard MD −0.54; 95% CI −1.94 to 0.85, *p* = 0.45, I^2^ 93%, *n* patients = 143). The two studies on SpA patients showed no efficacy of probiotics. Conclusions: Probiotic supplementation might decrease RA activity with a moderate decrease effect on CRP, but lack of evidence and studies’ heterogeneity do not allow us to propose them to patients with inflammatory arthritis to control their disease. Further RCTs are required in the future to determinate the efficacy of probiotics and the optimal administration design.

## 1. Introduction

The World Health Organization in 2002 defined probiotics as “living organisms in food and dietary supplements that upon ingestion can improve the health of the host beyond their inherent basic nutritional content” [1]. Naturally presents in fruits, raw vegetables, dairy products (in particular fermented ones), they are an integral part of the gut microbiota as a component of commensal flora. The main probiotic microorganisms used in human nutrition are lactic acid bacteria such as Lactobacillus and Bifidobacterium [2].

Compared with healthy people, patients with chronic inflammatory diseases, in particular rheumatoid arthritis (RA) and spondyloarthritis (SpA), have an altered gut microbiota called dysbiosis with an increased permeability allowing luminal antigens or bacteria to interact with the host immune system more readily [3,4]. This gut inflammation correlates with systemic inflammation and could be a trigger in developing some autoimmune diseases and participating in their severity [5,6,7,8].

Evidence from mice and human studies revealed that probiotics modulate locally and systemically the immune system, leading to a reduction in mucosal inflammation and pro-inflammatory cytokines [9]. They also alleviate joint inflammation in mice [10]. They can access the intestinal mucosal immune system, persist for a certain amount of time, and initiate a specific immune response. The interaction between probiotic strains and enterocytes is important for the controlled production of cytokines and chemokines secreted by epithelial cells. Indeed, it has been shown that some probiotic organisms can modulate the in vitro expression of pro- and anti-inflammatory molecules in a strain-dependent manner. Indeed, treatment with some Lactobacillus strains reduced gut permeability and decreased arthritic severity [11,12]. The impact of probiotics has already been well studied in atopic diseases and Crohn’s disease, for which they have not shown any real benefit.

Today, the growing interest of patients in the use of complementary therapies is justified by the existence of numerous side effects of usual disease-modifying anti-rheumatic drugs (DMARDS) and symptomatic treatments such as non-steroidal anti-inflammatory drugs (NSAIDs) and glucocorticoids (GCs). Probiotics could represent an alternative and complementary therapy to the standard drugs we already use to control rheumatic activity. However, the effect is not well documented in patients suffering from RA, SpA or psoriatic arthritis (PsA), especially recently, where studies are rare.

The available data from randomized controlled trials are highly heterogeneous in terms of the populations included, the characteristics of the rheumatism, and the results regarding activity scores and inflammatory markers. The three meta-analyses published attempted to provide conclusions on the efficacy of probiotics in patients with inflammatory rheumatism, with disease activity score in 28 joints (DAS28) and C-reactive protein (CRP) as the common primary endpoints. Two focused on RA, and the third included patients with SpA and juvenile idiopathic arthritis (JIA). The number and type of studies included as well as the population studied were therefore not homogeneous, and the results are discordant and difficult to interpret. The meta-analysis we performed is the first to pool only studies without high risk of bias and therefore to provide more conclusive results. It is also the first systematic review of the literature to examine the efficacy of probiotics in patients with SpA.

We aimed to provide a systematic literature review (SLR) of randomized controlled trials (RCT) and meta-analyses assessing the effect of probiotics on inflammatory rheumatic diseases’ symptoms and activity. This SLR was used to inform the recommendations of the French Society of Rheumatology (SFR) on diet in inflammatory rheumatic diseases [13].

## 2. Materials and Methods

This SLR of RCT and meta-analyses was conducted in accordance with the Preferred Reporting Items for Systematic Reviews and Meta-analyses (PRISMA) guidelines [14].

### 2.1. Search Strategy

Relevant articles were found using the MEDLINE database via PUBMED from inception to June 2020. A bibliography of selected retrieved articles was assembled and reviewed for inclusion by 2 researchers and a manual search was carried out. Conference abstracts from selected rheumatology meetings (European League Against Rheumatism (EULAR) and the American College of Rheumatology (ACR)) and from nutrition meetings (International Congress of Nutrition, European Nutrition Conference, American Society of Nutrition) from 2017 to 2019 were searched and manually reviewed for inclusion too.

Original research papers and reviews were searched using combinations of the grouped search terms: (“Spondylitis, Ankylosing”(Mesh) OR Spondylitis ankylosing OR ankylosis OR Spondylarthritis OR Spondylarthropathies OR “Arthritis, Rheumatoid”(Mesh) OR “rheumatoid arthritis” OR “rheumatoid” OR “Caplan Syndrome” OR “Felty Syndrome” OR “Rheumatoid Nodule” OR “Rheumatoid Vasculitis” OR “Arthritis, Psoriatic”(Mesh) OR “Psoriasis” OR “Arthritic Psoriasis” OR “Psoriatic Arthritis” OR “Psoriasis Arthropathica” OR “Psoriatic Arthropathy” OR “Arthropathies, Psoriatic” OR “Arthropathy, Psoriatic” OR “Psoriatic Arthropathies” OR “Spondylarthritis”(Mesh) OR “Spondylarthritides” OR “Spinal Arthritis” OR “Spinal Arthritides” OR “Arthritis, Spinal” OR “Spondyloarthritis”) AND (*Lactobacilli* OR *Lactobacillus* OR Bifidobacteria OR “Prebiotic” OR “Probiotic” OR “Synbiotic”).

Screening title and abstract took place initially followed by full-text screening. Search results were retrieved and duplicates were removed using Covidence software, V.1. developed by Veritas Health Innovation Ltd. (Melbourne, Australia). Disagreement was resolved by discussion and consensus between reviewers and senior researchers.

### 2.2. Eligibility Criteria

We included any open-label or blinded randomized controlled studies concerning adult patients with diagnosis of SpA, (PsA) or RA for whom an oral supplementation of probiotics was administered and disease activity assessed. This probiotic supplementation could be mixed with another diet in the intervention group. Control could be a placebo or another dietary intervention. We also included previous meta-analysis in our systematic literature review, to compare with ours and to make a synthesis of the available data.

We excluded any uncontrolled study, case reports, case series, letters, literature reviews, editorial comments, theses, reviews, book chapters, news, or isolated abstracts. We also excluded papers if their data were insufficient to be extracted or if not written in English or French.

Outcome measures included RA clinical activity markers such as disease activity score of 28 joints (DAS-28), EULAR or ACR response, Health Assessment Questionnaire Disability Index (HAQ), number of tender or swollen joints (TJC and SJC), visual analog scale (VAS) for disease activity by the patient, VAS for pain and global health score (GH score). Concerning SpA and PsA, outcomes were Bath Ankylosing Spondylitis Disease Activity Index (BASDAI), Bath Ankylosing Spondylitis Functional Index (BASFI), VAS for disease activity, quality of life (ASQoL), SJC, TJC, global well-being and bowel symptoms. Laboratory markers were C-reactive protein (CRP) and/or erythrocyte sedimentation rate (ESR). Disagreement in the determination of the eligibility of each study was resolved by consensus.

### 2.3. Data Extraction

Data of interest were collected from the included articles using the standardized extraction form. The extracted variables were publication date, journal, disease characteristics (including activity score, treatments such as DMARDs and symptomatic medications (GCs and NSAIDs), duration of the disease and positivity of rheumatoid factor (RF) and/or anti-citrullinated protein antibodies positive (ACPA)), study design, inclusion and non-inclusion criteria, probiotic strains, sample size, results in terms of disease activity, side effects and adherence. 

### 2.4. Quality Assessment

Risk of bias assessments used the Cochrane Collaboration tool for assessing risk of bias [15] and the Jadad scale [16]. Records limited to abstracts were not assessed because of the lack of information about study design. Two reviewers evaluated the quality of the studies independently (P.S. and C.D.).

### 2.5. Statistical Analysis

For the meta-analyses, the outcomes were the variation of DAS28 and CRP between inclusion and evaluation endpoint comparing the two groups of treatment (with or without probiotics). A narrative synthesis has been carried out to describe data extracted from articles that could not be included in the meta-analyses.

A standardized mean difference was estimated for DAS28 calculations due to heterogeneity in DAS28 scores across the included studies: some studies calculated the DAS28-ESR, and some calculated the DAS28-CRP. A mean difference (MD) was used for CRP calculations.

This provided a common weighted MD (or standardized MD) estimate with a 95% CI, taking into account weighting of the different samples. MD and 95% CIs are expressed as forest plots. Statistical heterogeneity of the selected studies was tested using the Q-test (χ^2^), applying a 0.05 statistical significance cutoff, and reported with the I^2^ statistic in which high values of I^2^, ranging from 0% to 100%, represent strong heterogeneity. In case of a significant heterogeneity, a random effect model was applied to take into account heterogeneity. All computations were performed using the RevMan V.5.3 software package developed by Nordic Cochrane Centre (Review Manager (computer program), V.5.3. Copenhagen, Denmark: The Nordic Cochrane Centre, the Cochrane Collaboration, 2011). *p* values lower than 0.05 were considered significant. Publication bias was checked through Egger’s test.

## 3. Results

### 3.1. Study Selection

**Figure 1:**
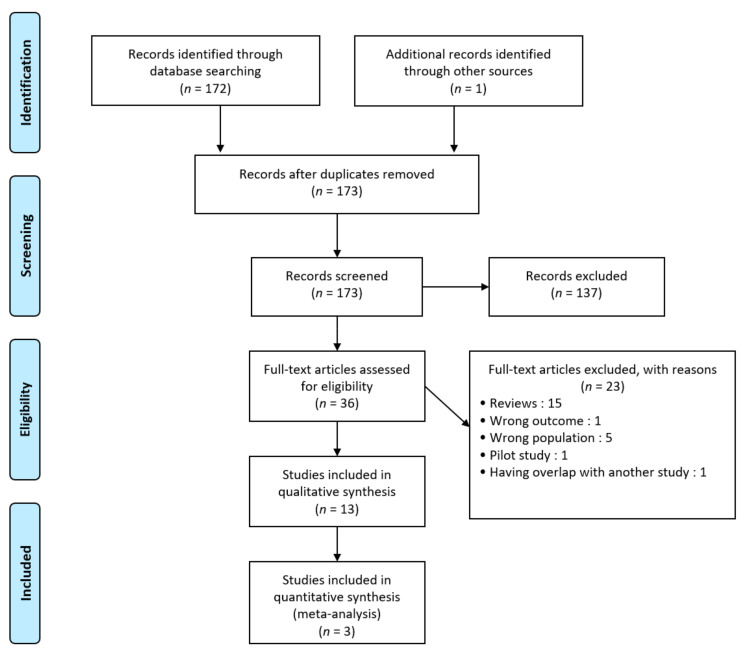
Preferred reporting items for systematic reviews and meta-analyses (PRISMA) diagram.

The literature review recognized a total of 172 records and one additional article was identified manually as shown in Figure 1. Of those, 137 were excluded after screening. Then, 23 studies were excluded after review because of wrong type of literature (*n* = 16), outcome (*n* = 1) or population (*n* = 5). One study was excluded because of the overlap with another study published by the same researchers at the same time [17]. Finally, 13 studies were included in the qualitative synthesis (including 10 RCTs and 3 meta-analyses) and 3 in our meta-analysis. No relevant unpublished studies were obtained from abstract meetings. 

### 3.2. Study Characteristics and Search Results

Table 1 presents the baseline characteristics of the included studies. All selected studies were published in English. Eight studies were conducted on patients with RA, 2 with SpA. Three meta-analysis about RA included some studies already selected but results about key variables concerned only a few of them. No RCT on patients with PsA was found.

Characteristics of individual studies are shown in Table 2. The total number of patients in the included RCT was 541 patients, 344 with RA, 196 with SpA and 1 patient with PsA (included in Jenks et al. probiotic group). Different strains of probiotics were evaluated, at different doses: 3 studies assessed oral supplementation with only one strain of *Lactobacillus* [18,19,20], 2 studies assessed a mix of two *Lactobacillus* strains [21,22], one study assessed *Bacillus coagulans* [23], the others assessed a mix of different probiotic types [24,25,26,27]. Time to assess response ranged from 8 weeks to 1 year. The comparator was placebo except in Vadell et al., which compares an anti-inflammatory diet rich in probiotics versus a typical Swedish diet, and in Nenonen et al., which compares an uncooked vegan diet rich in Lactobacilli versus a normal diet.

For RA, most studies assessed the efficacy on probiotic supplementation in reducing its activity in patients with active disease except one in which mean DAS-28 scores were below 2.6 [18]. Of note, three studies reported serum high-sensitivity CRP (hs-CRP) level change [18,24,25]. 

The outcome measurement corresponds to the date at which the primary and secondary endpoints were assessed, which also corresponds for all trials to the duration of probiotic or placebo/control supplementation.

The main inclusion criteria was RA diagnosed according to the 1987 ACR/EULAR criteria [18,19,24,25], other inclusion criteria were a disease duration of more than 1 year [18,19,23], more than 6 months for Zamani’s et al. and more than 2 years for Vadell et al. Treatments had to be at stable doses from 1 to 3 months prior to inclusion [18,19,20,21]. Two trials did not meet precisely the minimum activity score required [18,19] whereas all others referred to at least mild activity according to the DAS28.

Concerning SpA studies, patients were included only if they had a sacroiliitis (radiographic or magnetic resonance imaging (MRI)) in one of these studies [27]; the second one assessed patients with SpA without distinction on clinical phenotypes (i.e., axial, peripheral and/or enthesitic) [26]. 

Mean disease duration was between 4.7 and 19 years for RA and between 7.9 and 20.3 years for SpA. Little information was available concerning the proportion of patients taking targeted therapies; one study in RA specified the use of DMARD as a non-inclusion criteria [19] whereas another one in RA specified the use of NSAIDs as a non-inclusion criteria [18].

### 3.3. Risk of Bias within Studies

**Figure 2:**
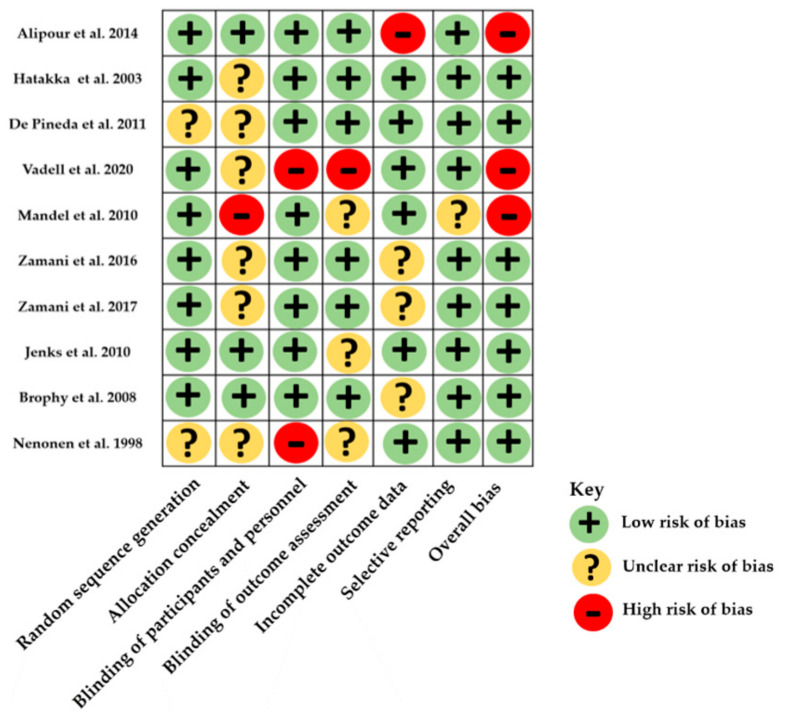
Distribution of risk-of-bias judgments within each bias domain of the Cochrane Collaboration tool.

The risk of bias assessment ranged from low to high and is illustrated in Figure 2 and Table S1 (Supplementary Materials). All studies were double-blinded except 2 single-blinded studies whose probiotics were not the unique intervention, thus had a high risk of bias with Cochrane’s tool and a Jadad score of 3 [20] and 2 [22]. Two other studies were considered to have a high risk of bias because of attrition bias [18] and inappropriate data reporting [23]. Most studies were monocentric. Two studies had a Jadad score of 5 [26,27]. 

### 3.4. Outcomes

#### 3.4.1. Results in Rheumatoid Arthritis

**Figure 3:**
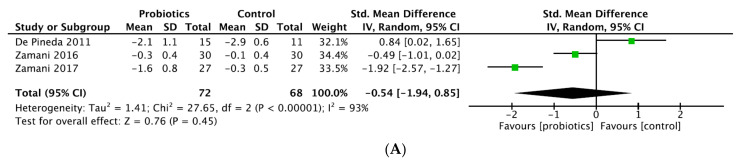

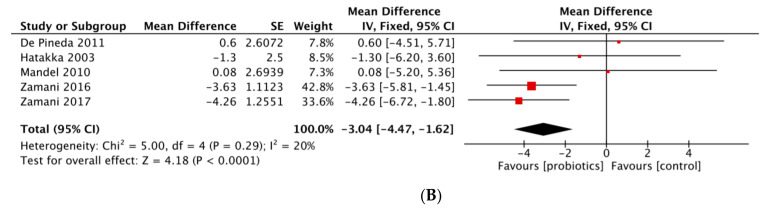
(**A**) Forest plot of disease activity score variations in rheumatoid arthritis. (**B**) Forest plot of CRP variations in rheumatoid arthritis.

Study outcomes are summarized in Table 3, sorted according to significant results in favor of probiotic supplementation or not. Results from former meta-analyses [28,29,30] are detailed in Table 4; data from our meta-analysis are shown in Figure 3.

- DAS28

Six studies provided data on DAS28 [18,20,21,22,24,25]. A significant improvement of DAS28 was achieved by three trials comparing probiotic supplementation with placebo [18,24,25]. On other hand, three trials concluded with no significant results [20,21,22], in particular Vadell et al. comparing an anti-inflammatory diet (rich in fatty acid, fibers and probiotics) versus typical Swedish diet, and Nenonen et al. comparing an uncooked vegan diet (fermented wheat drink, wheat grass drink, dietary fiber) with normal diet. The fact that probiotics supplementation was mixed with another dietary intervention in these studies may have been a confounding factor and altered the quality of the results because the efficacy of probiotics is difficult to individualize. Moreover, the cross-over design is questionable, especially because the washout period is arbitrary and does not allow to clearly establish that the first diet taken had no influence on the microbiota composition during the second period of the study (Table 3).

Our meta-analysis included three studies after exclusion of high-risk-of bias studies [18,20,22], and revealed probiotic supplementation did not show a significant effect in reducing DAS28-CRP with a SMD (95% CI) of −0.54 (−1.94 to 0.85) (*p* = 0.45; I^2^ = 93%; *n* patients = 143) (Figure 3A). Two former meta-analyses showed significant decrease of DAS28-CRP: Rudbane et al. (SMD (95% CI) −0.58 (−0.97 to −0.19), *p* = 0.94, I^2^ = 0%, *n* patients = 106), included only two RCT; and Lowe et al. (−0.28 (−0.5 to −0.05), *p* = 0.016) included four studies (sample size not reported) but compared post-treatment DAS28 values between groups, which is not appropriate as baseline DAS28 values in intervention group and comparators were clearly different in two studies [17,21] (Table 4). In contrast, the meta-analysis from Mohammed et al. concluded there was no significant effect with SMD (95% CI) 0.023 (−0.584 to 0.631), *p* = 0.94, I^2^ = 73%, *n* patients = 132, but included a high-risk-of-bias study [18]. *Lactobacillus* was the only strain used in all trials included, except Zamani RCTs, which pooled different probiotic strains.

- Inflammatory markers

We found eight studies provided data on CRP and/or ESR and 3 of them concerned hs-CRP (Table 3) [18,24,25]. In our SLR, three studies provided significant reduction of hs-CRP whereas results of four studies indicated no significant beneficial effects of probiotic supplementation on CRP [19,21,22,23]. No significant decrease concerning ESR was reported. Our meta-analysis pooled five RCTs and revealed a significant effect in reducing CRP with a MD (95% CI) of −3.04 mg/L (−4.47 to −1.62 mg/L) (*p* < 0.001) by analyzing a total of 209 patients (Figure 3B). The heterogeneity was moderate (*p* = 0.29; I^2^ = 20%). Meta-analysis from Mohammed et al. and Rudbane et al. did not show significant decrease of CRP (respectively, SMD (95% CI) −2.660 mg/L (−6.144 to 0.823), *p* = 0.134, I^2^ = 82%; SMD (95% CI) −0.27 mg/L (−0.77 to 0.23), *p* = NS, I^2^ = 55%), pooling 191 and 132 RA patients. The same results were obtained for ESR. Lowe et al., who combined seven studies, concluded there was a statistically significant reduction of CRP (SMD (95% CI) −2.34 mg/L (−4.26 to −0.41), *p* = 0.017, I^2^ = 52%). However, two trials with SpA and JIA patients were added and post-treatment values rather than variations were compared between intervention and comparator groups [26,31]. In the sub-population analysis on RA patients, no benefit of probiotics was found (Table 4).

- TJC

We found only one study out of seven with results in favor of probiotics supplementation (MD (95% CI) −0,72, (−1,19 to −0,25), *p* = 0.003) (Table 3) [18]. The two meta-analysis, with a total of 191 and 153 patients (Table 4) [29,30], did not show a significant difference between probiotic and placebo groups (respectively, SMD (95% CI) 0.379 (−0.578 to 1.336), *p* = 0.437, I^2^ = 71%; SMD (95% CI) −0.21 (−0.53 to 0.11), I^2^ = 10%). Mohammed et al. included a study with some JIA patients.

- SJC

We found only one study out of seven with results in favor of probiotics supplementation (MD (95% CI) −0.351, (−0,58 to −0,13), *p* = 0.003) (Table 3) [18]. Two meta-analysis available did not shown any significant difference between probiotic and placebo groups (respectively, SMD (95% CI) 0.171, (−0.391 to 0.733), *p* = 0.551, I^2^ = 54%; SMD (95% CI) −0.30 (0.62 to 0.02), I^2^ = 0%) by pooling 191 and 153 patients (Table 4) [29,30].

- HAQ

One study showed a significant improvement in the HAQ score in the probiotic group but no between-group differences (MD (95% CI) −0,2 (−0,5 to 0,1), *p* = 0,29) [19], one study showed a positive but not significant influence (MD −0.11, *p* = 0.11) [21] and one study did not show any significant effects (MD (95% CI) 0.006 (−0,33 to 0,35), *p* = 0.97) [23]. No significant HAQ improve was noticed in two meta-analysis (data not shown) [29,30]. 

#### 3.4.2. Results in Spondyloarthritis

Studies’ results are shown in Table S2 (Supplementary Materials). Two studies concluded no significant decrease in any disease activity markers. Intestinal chronic inflammatory disease was an exclusion criteria for Jenks et al. and about 75% of patients had been taking NSAIDs in both groups at baseline, which may have altered gut microbiota.

### 3.5. Tolerance Data

Five studies concerning RA reported the absence of any side effects related to probiotic supplementation [18,21,23,24,25]. One RCT did not report any information on this subject [19]. Vadell et al. described a 30% proportion of patients during the intervention periods who experienced moderate digestive intolerance such as nausea, diarrhea, stomach pain, heartburn and gas but no dropouts were related and most of them were present only at the start of a diet period.

Adverse events from spondyloarthritis RCTs were well documented: Vadell et al. mentioned the occurrence of nausea, increased flatus, diarrhea, vomiting and abdominal pain in the same proportion between the two groups. Brophy et al. reported 5 people in the placebo group and 6 in the probiotic group with adverse events, mainly stomach cramps (3) and indigestion (1) in both groups.

## 4. Discussion

This work updated the SLR and meta-analysis on probiotics in chronic inflammatory rheumatism and found: (1) a non-significant decrease of DAS28-CRP, (2) a significant decrease of CRP levels with RA patients under probiotics, (3) the absence of efficacy in patients with SpA. This work was used to inform the French Society of Rheumatology guidelines on diet in chronic inflammatory rheumatisms.

*Bifidobacterium* and *Lactobacillus* are known to provide many beneficial effects, in mice and humans. A recent meta-analysis in 2020 reported significant effects in the reduction of blood pressure, lipid profile, BMI and serum glucose [32]. Other evidence has shown their power of reducing intestinal permeability and modulating immune function through direct interaction with the mucosal immune system [33,34]. Immunological studies revealed that probiotics have dose and duration-dependent immunomodulatory effects on B and T cell proliferation and affect pro-inflammatory and anti-inflammatory cytokine regulation [35]. Probiotics administration can restore the normal mucosal barrier function through keeping the balance between intestinal microflora and resistance against harmful bacterial colonization, adherence and translocation [36]. These effects are dependent on the species and strain of bacteria [37]. For instance, oral administration of *Lactobacillus rhamnosus* attenuates various types of experimental arthritis [38]. Increasing evidence suggests that gut dysbiosis in RA and SpA patients favors inflammation, participating in disease activity and severity [39,40]. However, the data on gut microbiota composition are controversial and the strains contained in probiotics are not specifically decreased. 

Concerning RA, although a statistically significant decrease in CRP was identified, cautious interpretation is required before inferring clinical significance. First, the reduction in CRP with a MD (95% CI) of −3.04 mg/L (−4.47 to −1.62 mg/L) may not represent a clinically meaningful change. On the other hand, some studies reported normal baseline values of both CRP and ESR, but they did not provide the data and it could not be extracted a posteriori. That may partially explain the absence of significant results regarding DAS28, of which inflammatory markers are one of the components. This systematic review of the literature provided very different baseline characteristics and inclusion criteria for both RA and SpA patients; e.g., only women were included in Alipour et al. [18], the minimum duration of disease progression at inclusion was variable, it had to be either more than 6 months for Zamani’s et al., more than 2 years for Vadell et al., one year for the others or unspecified. Despite this, the average disease duration was more than 5 years in all studies, provided longstanding RA and SpA. Therefore, as studies did not provide newly diagnosed RA and SpA, the results cannot be extrapolated to this population. Another limitation of this meta-analysis is the small sample size, which may affect the reliability and validity of the results. Besides, four studies were monocentric [18,20,22,23], once again limiting the generalization of the results. Those are important limitations to propose the routine use of probiotics in patients with RA.

Concerning DAS28, three studies showed a significant improvement comparing probiotic supplementation with placebo, but results concerning TJC and SJC are not clinically pertinent [18,24,25]. In fact, in Alipour et al., patients in both groups had no or few TJC or SJC. It is important to note that studies included in meta-analyses for evaluation of DAS28, TJC, SJC, CRP, ESR and HAQ are different, which affects the comparability of the results. Furthermore, the control group was different with two studies comparing probiotics with another dietary intervention, no significant effect on DAS-28, CRP, ESR, TJC and SJC was observed in these trials, suggesting a potential confusion bias. Finally, we may suggest that it is not surprising that meta-analysis of Rudbane et al. reports a significant improvement of DAS28 by the fact that the only two studies which reported significant findings were included.

Concerning meta-analyses, as a primary outcome, we analyzed the variation of the values before and after supplementation, as Mohammed et al. and Rudbane et al. did, whereas Lowe et al., who reported significant results, compared final values between each group. This limits their results, as values before intervention were not the same. The strengths of our meta-analysis are that we compared variations in outcome measures (such as DAS28 and CRP) between the two groups and included only studies which were RCTs with low or moderate risk of bias and homogeneous disease populations and added new studies. We were interested in three types of rheumatism because they are the focus of most of the literature data currently available on probiotics and represent the largest proportion of patients with inflammatory rheumatic disease. Concerning DAS28 and CRP, Alipour et al. was excluded from our analysis as the results were not reported in intention to treat. In Vadell et al. and Nenonen et al., an additional dietary intervention was performed which could influence the results, motivating us to exclude them from the meta-analysis. Inclusion of Alipour et al. did not change the results for DAS-28 (SMD (95% CI) of −0.59 (−1.55 to 0.37), *p* = 0.23; I^2^ = 89%; *n* patients = 189) (data not shown). Furthermore, inulin supplementation (prebiotic) in Zamani et al., 2017 may allow better engraftment of probiotics in the gastrointestinal tract compared to isolated probiotics. 

Another strength of our meta-analysis is its methodological quality. We used the “A MeaSurement Tool for Assessing systematic Reviews” (AMSTAR2), designed to carry out rapid and reproducible assessments of the quality of conduct of systematic reviews of RCTs. It represents one of the most widely used instruments to date. Analysis of the methodological quality according to this tool revealed that it was critically low for the three previous meta-analyses. Ours had the advantage of being of higher quality because we removed RCTs at high risk of bias and conducted an adequate investigation of publication bias.

The results of Egger’s test revealed the absence of publication bias for DAS28 (*p* value = 0.96) and a publication bias for CRP (*p* value = 0.02). This bias disappeared when sensitivity analyses were performed by removing the studies of Pineda et al. (*p* value = 0.07) or Mandel et al. (*p* value = 0.08) (data not shown). Ours to date is the only meta-analysis clearly describing publication bias regarding probiotics supplementation in RA, from which it is possible to conclude the reliability of the results regarding DAS28. The search for publication bias was theoretically limited by the small number of RCTs available in the literature, but our search strategy was intended to be as complete as possible, including searching for conference abstract data. The number of RCTs included is consistent with previous meta-analyses or higher. This limits the risk of not having included relevant RCTs.

Regarding spondyloarthritis, only two RCTs assessed the efficacy of probiotics supplementation versus placebo on SpA activity. No significant decrease of activity score or well-being was found. It is important to note the disparity between the type of patients included, from peripheral phenotype to ankylosing spondylitis and sacroiliitis confirmed by MRI. It would have been interesting to evaluate the effect of probiotic supplementation in a more homogeneous group of patients because these phenotype differences may have affected the final results. More studies are needed to assess the efficacy of probiotics in this selected population.

Tolerance data are reassuring; however, adverse events were not primary endpoints, therefore this data may have suffered from a lack of collection and precision in its measurement. All studies reported an excellent observance except that of Brophy et al., which suffered from less than 70% of tablets being taken.

Furthermore, it has been shown that drugs influence the intestinal microbiota composition and as such might impact response to probiotics. At baseline, in general, stable antirheumatic medication between one and three months before inclusion were one of the inclusion criteria in RA studies. Almost all patients with RA appeared to be treated with either DMARD or symptomatic treatments such as glucocorticoids or NSAIDs. Only two studies specify the proportion of patients not taking any medication, that of Mandel et al. where it was equal to 6% in the probiotic group (*n* = 1/15) and 7% in the control group (*n* = 1/14), and Pineda et al. with 4% in the probiotic group (*n* = 1/23) and 13.6% in the control group (*n* = 3/22). However, most of the studies did not mention whether any modification of medication occurred during follow-up, especially biological DMARD (bDMARD), corticosteroids and NSAIDs use, which would be important confounding factors. Disparity in publication years also influence the medications taken by patients, in particular bDMARD. Baseline rheumatism activities were also very different between studies, which influences microbiota composition and possibility of activity variations with intervention. 

It is currently difficult to conclude whether probiotics are efficient or not because of a high heterogeneity in studies’ design due to the use of different strains, quantities and duration of supplementation. In regard to the heterogeneity present between the RCTs, a large heterogeneity exists between the previous meta-analyses, which can explain the differences in their results concerning the DAS28 and the CRP. Indeed, the main limitation is the wide variety of probiotic strains, administration dose and duration among studies with insufficient power to perform subgroup analysis. No study argued for the dosage of probiotics based on a possible pathophysiological rationale or evidence-based medicine. In addition, strains were not adapted to the profile of the patient’s initial microbiota; if they were targeted to a possibly deficient one it would have been interesting to see if the effect was significantly increased, which is currently analyzed with the new generation probiotics. 

In addition to the limitations of the literature studied, our systematic review may have been impacted by the fact that the initial screening for inclusion and exclusion was undertaken by two reviewers, introducing the risk of human error. 

In summary, according to the available evidence, a literature review provided data on probiotic supplementation in patients with established rheumatism and low markers of inflammation, impacting the generalizability of the results. There has been shown to be a significant decrease of CRP concerning RA patients, but clinical relevance is not demonstrated. Probiotic supplementation might decrease RA activity but lack of evidence and heterogeneity lead us to conclude they should not be proposed to patients with inflammatory arthritis to control their disease. However, the good tolerance and potential cardiovascular benefits should be noted and patients who are willing to take them should be informed but not discouraged. Further RCTs and meta-analysis are required in the future to determinate the efficacy of probiotics and in which administration schema. 

## Figures and Tables

**Table 1:** Baseline characteristics of patients included in the RCTs.

Study	Country	Inclusion Criteria	Groups	Age (Years) Mean (SD)	Disease Duration (Years) Mean (SD)	RF + N (%)	ACPA + N (%)	Activity Score Mean (SD)	Current Medication			
									csDMARDs N (%)	bDMARDs N (%)	Oral CS N (%)	NSAIDs N (%)
Rheumatoid arthritis: *n* = 8												
Alipour et al., 2014 [18]	Iran	Women, ACR 1987, for at least 1 year, DAS-28 CRP < 5.1, 20–80 years, no NSAIDs or bDMARDs, oral CS < 10 mg/day	Probiotics	41.14 (12.65)	5.25 (3.75, 10.0) ^a^	NR	NR	DAS-CRP 2.56 (1.01)	HCQ: 18 (81.8)	0	21 (95.5)	NR
			Placebo	44.29 (9.77)	4.75 (3.0, 9.0) ^a^			2.31 (0.90)	MTX: 20 (83.3)	0	23 (95.8)	NR
Hatakka et al., 2003 [19]	Finland	ACR 1987, 18–64 years, for at least 1 year, no DMARDs, oral CS < 7.5 mg/day	Probiotics	50 (10)	8.3 (7.3)	5 (62.5)	NR	NR	0	0	6 (75)	6 (75)
			Placebo	53 (7)	11.0 (8.2)	7 (53.8)			0	0	8 (62)	10 (77)
Vadell et al., 2020 [20]	Sweden	18–70 years, for at least 2 years, DAS-28 ESR ≥ 2.6	Probiotics mixed with diet rich in fatty acids and fibers	61 (12) ^b^	20.0 (9.5) ^b^	34 (72) ^c^		DAS-ESR 3.8 (0.9)	MTX: 31 (66) ^b^	16 (34) ^b^	12 (26) ^b^	24 (51) ^b^
			Typical Swedish diet					3.6 (0.8)				
Pineda et al., 2011 [21]	Canada	ACR criteria, 18–80 years, SJC and TJC ≥ 4, no intra-articular CS ≤ 1 month before	Probiotics	63.8 (7.5)	19 (12.4)	NR	NR	DAS-CRP 4.18 (1.05)	MTX: 11 (73)	NR	4 (26)	NR
			Placebo	59.1 (9.1)	13.7 (8.4)			4.83 (0.91)	MTX: 11 (78)		3 (21)	NR
Nenonen et al., 1998 [22]	Finland	SJC > 3 or TJC > 5, ESR > 20 mm/h or CRP > 10 mg/L	Probiotics with uncooked vegan diet	49.1 (7.1)	12.6 (10.3)	15 (79) ^c^		DAS-CRP 3.26	MTX: 10 (52.6)	NA	10 (52.6)	16 (84.2)
			Normal diet	55.6 (10.8)	16.1 (13.6)	14 (70%) ^c^		3.44	MTX: 5 (25)		9 (45)	18 (90)
Mandel et al., 2010 [23]	USA	18–80 years, for at least 1 year, oral CS < 10 mg/day, four or more among: MS ≥ 1 h, STS in ≥ 3 joint areas, swelling of IPP or MCP or wrist joints, rheumatoid nodules, FR+, erosions	Probiotics	NR	NR	NR	NR	NR	18 (78) ^d^ 17 (77) ^d^		NR	2 (9.1)
			Placebo									3 (13.6)
Zamani et al., 2016 [24]	Iran	ACR 1987, 25–70 years, for at least 6 months, DAS-28 CRP > 3.2, no bDMARDs	Probiotics	52.2 (12.2)	7.0 (5.7)	NR	NR	DAS-CRP 4.0 (0.7)	MTX: 29 (96.7)	0	27 (90.0)	NR
			Placebo	50.6 (13.1)	7.0 (6.7)			4.1 (0.7)	MTX: 29 (96.7)	0	28 (93.3)	
Zamani et al., 2017 [25]	Iran	ACR 1987, 25–70 years, for at least 6 months, DAS-28 CRP > 3.2, no bDMARDs	Probiotics	49.3 (11.0)	7.7 (6.1)	NR	NR	DAS-CRP 4.2 (0.7)	MTX: 26 (96.3)	0	24 (88.9)	NR
			Placebo	49.5 (12.9)	7.5 (6.4)			3.5 (0.8)	MTX: 26 (96.3)	0	25 (92.6)	
Spondyloarthritis: *n* = 2												
Jenks et al., 2010 [26]	New Zealand	ESSG criteria, more than 18 years, BASDAI ≥ 3, BASFI ≥ 3, MASES ≥ 3, TJC or SJC ≥ 2	Probiotics	45.5 (15)	9.8 (13)	NR	NR	BASDAI 4.2 (2.2)	MTX: 2 (6)	NR	0	24 (75)
			Placebo	41.1 (10)	7.9 (7)			4.5 (2.0)	MTX: 3 (10)		2 (7)	24 (77)
Brophy et al., 2008 [27]	UK	X-ray or MRI sacro-ilitis, more than 18 years	Probiotics	44.8 (12.1)	20.3 (13.2)	NR	NR	NR	5 (7.9) d		0	53 (85.5)
			Placebo	42.7 (12.7)	20.3 (13.4)				8 (11.9) d		2 (3.0)	44 (66.7)

Age and disease duration are presented as mean and standard deviation (SD). Current medications are presented as number and percentage (%). ^a^ Data are presented as median (percentiles 25 and 75) ^b^ Participants who completed ≥1 diet period; ^c^ ACPA and/or RF positive; ^d^ DMARDs in general. ACR: American College of Rheumatology; ACPA: Anti-citrullinated Protein/Peptide Antibodies; BASDAI: Bath Ankylosing Spondylitis Disease Activity Index; BASFI: Bath AS Functional Index; bDMARDs: biological Disease Modifying AntiRheumatic Drug; CS: corticosteroid; csDMARDs: conventional synthetic; CRP: C-reactive Protein; DAS-28: Disease Activity Score in 28 joints; ESR: Erythrocyte Sedimentation Rate; ESSG: European Spondyloarthropathy Study Group; HCQ: Hydroxychloroquine, IPP: InterPhalangeal Proximal; MASES: Maastrich Ankylosing Spondylitis Enthesitis Score; MCP: MetaCarpoPhalangeal; MS: Morning Stiffness; MRI: Magnetic Resonance Imaging; MTX: Methotrexate; NA: Not Available; NR: Not Reported; NSAIDs: Non Steroidal Anti-Inflammatory Drugs; RCT: Randomized Controlled Trial; RF: Rheumatoid Factor; SD: Standard Deviation; SJC: Swollen Joint Count; STS: Soft Tissue Swelling; TJC: Tender Joint Count; USA: United States of America; UK: United Kingdom.

**Table 2:** Study characteristics of the 10 studies included in the systematic review sorted by probiotic type.

Study	Disease	Probiotic Strains	Other Intervention	Design	Population	Intervention		Control		Outcome	Outcome Measurement
						Type	N	Type	N		
***Lactobacillus*: n = 5**											
Alipour et al., 2014 [18]	RA	*L. casei* 01	No	Double-blind RCT	46	10^8^ CFU (capsule) daily for 8 weeks	22	Placebo	24	DAS-28 CRP, SJC, TJC, GH score, hs-CRP, moderate EULAR response	8 weeks
Hatakka et al., 2003 [19]	RA	*L.rhamnosus* GG, ATCC 53103	No	Double-blind RCT	21	≥5 × 10^9^ CFU (capsule) twice daily for 1 year	8	Placebo	13	SJC, TJC, HAQ score, ESR, CRP, VAS activity	1 year
Pineda et al., 2011 [21]	RA	*L.rhamnosus* GR1 and *L.reuteri* RC-14	No	Double-blind RCT	29	2 × 10^9^ CFU (capsule), each twice daily for 3 months	15	Placebo	14	ACR20 response, DAS-28 CRP, SJC, TJC, MS, HAQ score, ESR, CRP, VAS pain, VAS fatigue	3 months
Nenonen et al., 1998 [22]	RA	*L. plantarum and* *L. brevis*	Uncooked vegan diet	Single-blind RCT	39	Daily “living food” diet in packed form containing fermented wheat drink rich in Lactobacilli	19	Normal diet	20	DAS-28 ESR, CRP, ESR, TJC, SJC, HAQ, MS, VAS pain	3 months
Vadell et al., 2020 [20]	RA	*L.plantarum* 299 v	Anti-inflammatory diet (rich in fatty acids and fibers): fish, vegetables, cereals	Single-blind crossover RCT	50	One shot 5 days a week for 10 weeks	26 ^a^	Typical Swedish diet	24 ^a^	DAS-28 CRP, DAS-28 ESR, SJC, TJC, ESR, GH score	10 weeks
***Bacillus***: **n = 1**											
Mandel et al., 2010 [23]	RA	*Bacillus coagulans*	No	Double-blind RCT	45	2 × 10^9^ CFU (capsule) daily for 2 months	23	Placebo	22	ACR20 response, SJC, TJC, HAQ score, VAS pain, VAS activity, ESR, CRP	2 months
**Mix of different probiotics types: n = 4**											
Zamani et al., 2016 [24]	RA	*L. acidophilus,* *L.casei* and *Bifidobacterium bifidum*	No	Double-blind RCT	60	2 × 10^9^ CFU/g (capsule) each strain, daily for 2 months	30	Placebo	30	DAS-28 CRP, SJC, TJC, hs-CRP, VAS pain	2 months
Zamani et al., 2017 [25]	RA	*L. acidophilus,* *L. casei* and *Bifidobacterium bifidum*	Prebiotic inulin 800 mg	Double-blind RCT	54	2 × 10^9^ CFU/g (capsule) each strain, daily for 2 months	27	Placebo	27	DAS-28 CRP, SJC, TJC, hs-CRP, VAS pain	2 months
Jenks et al., 2010 [26]	SpA	*Streptococcus salivarius* K12, *Bifidobacterium lactis* LAFTI B94 and *L. acidophilus* LAFTI L10	No	Double-blind RCT	63	10^8^ CFU/g, 4 × 10^8^ CFU/g, and 4 × 10^8^ CFU/g (powder, about 0.8 g in total twice daily) for 3 months	32	Placebo	31	BASFI10 response, BASDAI, ASAS20, VAS pain, fatigue, ASQoL, SJC, TJC, CRP	3 months
Brophy et al., 2008 [27]	SpA	*L. salivarius* CUL61*, L. paracasei* CUL08*, Bifidobacterium infantis* CUL34 and *Bifidobacterium bifidum* CUL20	No	Double-blind RCT	134	6.25 × 10^9^ CFU, 1.25 × 10^9^ CFU, 1.25 × 10^9^ CFU and 1.25 × 10^9^ CFU (capsule) daily for 3 months	65	Placebo	69	VAS activity, global well-being, bowel symptoms	3 months

^a^ Patients who started first by intervention or control diet. ACR: American College of Rheumatology; ASAS: Assessment of Spondyloarthritis International Society; ASQoL: Ankylosing Spondylitis Quality of Life; BASDAI: Bath Ankylosing Spondylitis Disease Activity Index; BASFI: Bath Ankylosing Spondylitis Functional Index; bDMARDs: biological Disease Modifying AntiRheumatic Drug; CFU: Colony-Forming Unit; CS: Corticosteroid; csDMARDs: conventional synthetic Disease Modifying AntiRheumatic Drug; hs-CRP: high sensitivity C-reactive Protein; DAS-28: Disease Activity Score in 28 joints; ESR: Erythrocyte Sedimentation Rate; EULAR: European League Against Rheumatism; GH: Global Health; HAQ: Health Assessment Questionnaire; L.: *Lactobacillus*; MS: Morning Stiffness; NR: Not Reported; NSAIDs: NonSteroidal Anti-Inflammatory Drugs; RA: Rheumatoid Arthritis; RCT: Randomized Controlled Trial; SD: Standard Deviation; SJC: Swollen Joint Count; SpA: Spondyloarthritis; TJC: Tender Joint Count; VAS: Visual Analogic Scale.

**Table 3:** Study results sorted by covariates in rheumatoid arthritis.

Study		Outcome	Intervention	Control	Mean Difference between Groups *	*p*-Value (Intervention vs. Controls)
			Baseline Versus End of Treatment	Baseline Versus End of Treatment		
DAS28						
In favor probiotic intervention	Alipour et al., 2014 [18]	DAS28-CRP	2.56 (1.01) vs. 2.07 (0.82)	2.31 (0.90) vs. 2.23 (0.86)	−0.31 (−0.61; −0.02)	*p* = 0.039
	Zamani et al., 2016 [24]	DAS28-CRP	4.0 (0.7) vs. 3.7 (0.7)	4.1 (0.7) vs. 4.0 (0.7)	−0.2	*p* = 0.01
	Zamani et al., 2017 [25]	DAS28-CRP	4.2 (0.7) vs. 2.6 (0.7)	3.5 (0.8) vs. 3.2 (1.1)	−1.3	*p* < 0.001
No significant result	Pineda et al., 2011 [21]	DAS28-CRP	−2.1 (1.1) ^a^	−2.9 (0.6) ^a^	0.8	*p* = 0.77
	Vadell et al., 2020 [20]	DAS28-CRP	−0.455 (−0.698; −0.212) ^b^	−0.222 (−0.461; 0.017) ^b^	−0.233 (−0.569; 0.103)	*p* = 0.169
		DAS28-ESR	−0.369 (−0.628; −0.111) ^b^	−0.080 (−0.335; 0.174) ^b^	−0.289 (−0.652; 0.075)	*p* = 0.116
	Nenonen et al., 1998 [22]	DAS28-ESR	3.26 vs. 3.01	3.44 vs. 3.46	−0.23	*p* = 0.7
Inflammatory markers						
In favor probiotic intervention	Alipour et al., 2014 [18]	hs-CRP (mg/L)	3.10 (1.32; 18.01) vs. 2.80 (0.95; 15.95) ^c^	2.30 (1.23; 7.99) vs. 3.50 (0.89; 10.38) ^c^	−2.03 (−3.51; −0.54)	*p* = 0.009
	Zamani et al., 2016 [24]	hs-CRP (mg/L)	7.27 (6.24) vs. 6.61 (6.03)	6.02 (5.78) vs. 9.09 (7.46)	−3.73	*p* < 0.001
	Zamani et al., 2017 [25]	hs-CRP (mg/L)	6.0·0 (4.8) vs. 4.6 (2.7)	5.6 (5.1) vs. 8.5 (6.8)	−4.3	*p* = 0.001
No significant result	Hatakka et al., 2003 [19]	CRP (mg/L)	1.6 (4.6) vs. 2.6 (3.3)	5.1 (5.7) vs. 7.4 (8.7)	−1.3 (−6.2; 3.6)	*p* = 0.582
		ESR (mm/h)	17.3 (14.7) vs. 20.7 (17.3)	18.2 (15.9) vs. 17.9 (14.4)	3.6 (−0.7; 7.9)	*p* = 0.095
	Mandel et al., 2010 [23]	CRP (mg/L)	NR	NR	0.008 (−0.52. 0.53)	*p* = 0.98
		ESR (mm/h)	NR	NR	−0.054 (−0.49. 0.38)	*p* = 0.80
	Pineda et al., 2011 [21]	CRP (mg/L)	1.8 (8.4) ^a^	1.2 (4.8) ^a^	0.6	*p* = 0.75
		ESR (mm/h)	−4.0 (9.8) ^a^	0.27 (6.8) ^a^	−4.27	*p* = 0.76
	Vadell et al., 2020 [20]	ESR (mm/h)	−0.051 (−0.347; 0.245) ^b^	0.210 (−0.081; 0.501) ^b^	−0.261 (−0.661; 0.138)	*p* = 0.194
	Nenonen et al., 1998 [22]	CRP (mg/L)	NR	NR	NR	*p* = NS
		ESR (mm/h)				
TJC						
In favor probiotic intervention	Alipour et al., 2014 [18]	TJC	0.0 (0.0; 2.25) vs. 0.0 (0.0; 1.0) ^c^	0.0 (0.0; 2.75) vs. 0.0 (0.0; 2.75) ^c^	−0.72 (−1.19; −0.25)	*p* = 0.003
No significant result	Hatakka et al., 2003 [19]	TJC	3.7 (2.5) vs. 2.5 (1.7)	3.0 (3.3) vs. 2.6 (2.4)	−0.3 (−2.2; 1.7)	*p* = 0.784
	Mandel et al., 2010 [23]	TJC	NR	NR	−0.074 (−0.81. 0.66)	*p* = 0.84
	Pineda et al., 2011 [21]	TJC	0.2 (5.5) ^a^	−0.55 (7.1) ^a^	1.05	*p* = 0.43
	Zamani et al., 2016 [24]	TJC	5.2 (2.8) vs. 4.8 (2.6)	5.2 (2.5) vs. 4.7 (2.4)	0	*p* = 0.1
	Vadell et al., 2020 [20]	TJC	33.2 (16.1; 56.2) ^b^	27.1 (12.7; 48.7) ^b^	6.1 (−15.2; 27.3)	*p* = 0.572
	Nenonen et al., 1998 [22]	TJC	NR	NR	NR	*p* = NS
SJC						
In favor probiotic intervention	Alipour et al., 2014 [18]	SJC	0.0 (0.0; 2.0) vs. 0.0 (0.0; 1.0) ^c^	1.0 (0.0; 1.75) vs. 1.0 (0.0; 1.75) ^c^	−0.351 (−0.58; −0.13)	*p* = 0.003
No significant result	Hatakka et al., 2003 [19]	SJC	4.5 (5.5) vs. 2.1 (1.7)	2.5 (3.0) vs. 2.2 (3.1)	−1.1 (−3.0; 0.9)	*p* = 0.265
	Mandel et al., 2010 [23]	SJC	NR	NR	0.011 (−0.62. 0.64)	*p* = 0.97
	Pineda et al., 2011 [21]	SJC	−0.4 (3.3) ^a^	−1.0 (3.6) ^a^	0.6	*p* = 0.47
	Zamani et al., 2016 [24]	SJC	5.5 (3.0) vs. 5.1 (3.1)	5.8 (2.7) vs. 5.8 (2.8)	−0.37	*p* = 0.16
	Vadell et al., 2020 [20]	SJC	48.6 (23.8; 74.1) ^b^	37.3 (16.2; 64.5) ^b^	11.4 (−14.4; 37.2)	*p* = 0.383
	Nenonen et al., 1998 [22]	SJC	NR	NR	NR	*p* = NS

Data are presented as the mean (standard deviation) except contrary mention. * Difference between intervention group and placebo group (95% CIs); ^a^ Mean change from baseline to end of treatment (standard deviation); ^b^ Mean change from baseline to end of treatment (95% CIs); ^c^ Data presented as median (percentiles 25 and 75). hs-CRP: high sensitivity C-reactive Protein; DAS-28: Disease Activity Score in 28 joints; ESR: Erythrocyte Sedimentation Rate; NA: Not Applicable; NR: Not Reported; NS: Not Significant; SJC: Swollen Joint Count; TJC: Tender Joint Count.

**Table 4:** Meta-analysis available in rheumatoid arthritis and spondyloarthritis.

Meta-Analysis	Mohammed et al., 2017 [30]	Rudbane et al., 2018 [29]	Lowe et al., 2020 [28]
Method of results analysis	Comparison of Pre/post value variation	Comparison of Pre/post value variation	Comparison of final values
Methodological quality according to AMSTAR2 tool	Critically low	Critically low	Critically low
DAS28 CRP			
Studies included	Pineda et al., 2011 [21] Alipour et al., 2014 [18] Zamani et al., 2016 [24]	Alipour et al., 2014 [18] Zamani et al., 2016 [24]	Pineda et al., 2011 [21] Alipour et al., 2014 [18] Zamani et al., 2016 [24] Zamani et al., 2017 [25]
Total sample size	132	106	NR
Results	SMD = 0.023 (−0.584 to 0.631) *p* = 0.94 I^2^ = 73, *p* = 0.025	SMD = −0.58 (−0.97 to −0.19) *p* = NR I^2^ = 0.0, *p* = 0.634	SMD = −0.28 (−0.5 to −0.05) *p* = 0.016 I^2^ = NR
CRP			
Studies included	5 (NR)	Hatakka et al., 2003 [19] Pineda et al., 2011 [21] Alipour et al., 2014 [18] Zamani et al., 2016 [24]	Hatakka et al., 2003 [19] Pineda et al., 2011 [21] Alipour et al., 2014 [18] Zamani et al., 2016 [24] Zamani et al., 2017 [25] Jenks et al., 2010 [26] Shukla et al., 2016 [31]
Total sample size	191	132	NR
Results (mg/L)	SMD = −2.660 (−6.144 to 0.823) *p* = 0.134 I^2^ = 82.3, *p* < 0.001	SMD = −0.27 (−0.77 to 0.23) *p* = NS I^2^ = 55.3, *p* = 0.082	SMD = −2.34 (−4.26 to −0.41) *p* = 0.017 I^2^ = 52, *p* = 0.049
ESR			
Studies included	4 (NR)	Hatakka et al., 2003 [19] Pineda et al., 2011 [21]	-
Total sample size	129	47	-
Results (mm/h)	SMD = 1.861 (−4.481 to 8.202) *p* = 0.565 I^2^ = 66.0, *p* = 0.032	SMD = −0.17 (−0.76 to 0.42) *p* = NS I^2^ = 31.5, *p* = 0.0227	-
TJC			
Studies included	Hatakka et al., 2003 [19] Mandel et al., 2010 [23] Pineda et al., 2011 [21] Zamani et al., 2016 [24] Shukla et al., 2016 [31]	Hatakka et al., 2003 [19] Pineda et al., 2011 [21] Alipour et al., 2014 [18] Zamani et al., 2016 [24]	-
Total sample size	191	153	-
Results	SMD = 0.379 (−0.578 to 1.336) *p* = 0.437 I^2^ = 71.5, *p* = 0.007	SMD = −0.21 (−0.53 to 0.11) *p* = NS I^2^ = 10.1, *p* = 0.342	-
SJC			
Studies included	Hatakka et al., 2003 [19] Mandel et al., 2010 [23] Pineda et al., 2011 [21] Zamani et al., 2016 [24] Shukla et al., 2016 [31]	Hatakka et al., 2003 [19] Pineda et al., 2011 [21] Alipour et al., 2014 [18] Zamani et al., 2016 [24]	-
Total sample size	191	153	-
Results	SMD = 0.171 (−0.391 to 0.733) *p* = 0.551 I^2^ = 53.9, *p* = 0.07	SMD = −0.30 (0.62 to 0.02) *p* = NS I^2^ = 0.0, *p* = 0.462	-
VAS pain			
Studies included	-	-	Pineda et al., 2011 [21] Zamani et al., 2016 [24] Zamani et al., 2017 [25] Jenks et al., 2010 [26]
Total sample size	-	-	NR
Results	-	-	SMD = −8.97 (−15.38 to −2.56) *p* = 0.006 I^2^ = 41, *p* = 0.167

AMSTAR2: A MeaSurement Tool to Assess systematic Reviews version 2; CRP: C-Reactive Protein; DAS-28: Disease Activity Score in 28 joints; ESR: Erythrocyte Sedimentation Rate; NR: Not Reported; NS: Not significant; SJC: Swollen Joint Count; SMD: Standardized Mean Difference; TJC: Tender Joint Count; VAS: Visual Analogic Scale.

## Supplementary Materials

The following are available online at https://www.mdpi.com/article/10.3390/nu14020354/s1: Table S1: Quality assessment based on Jadad score of randomized controlled trials reviewed. Table S2: Study results in spondylarthritis.
