# Supplementary material for: Efficacy of Probiotics in Rheumatoid Arthritis and Spondyloarthritis: A Systematic Review and Meta-Analysis of Randomized Controlled Trials
Source: Nutrients. 2022 Jan 14;14(2):354. doi: 10.3390/nu14020354 (PMC8779560; doi:10.3390/nu14020354)
Supplement: Supplementary file 1 [file nutrients-14-00354-s001.zip › nutrients-1529562-supplementary.pdf]

Table S1. Quality assessment based on Jadad score of randomized controlled trials reviewed.

Study	Randomization	Blinding	Account of All Patients	Total
Alipour et al., 2014 [18]	1	2	1	4
Hatakka et al., 2003 [19]	2	1	1	4
Pineda et al., 2011 [21]	1	2	1	4
Vadell et al., 2020 [20]	2	0	1	3
Mandel et al., 2010 [23]	2	1	1	4
Zamani et al., 2016 [24]	2	1	1	4
Zamani et al., 2017 [25]	2	1	1	4
Jenks et al., 2010 [26]	2	2	1	5
Brophy et al., 2008 [27]	2	2	1	5
Nenonen et al., 1998 [22]	1	0	1	2

Table S2. Study results in spondylarthritis.

Study	Outcome	Intervention	Control	Mean Difference between Groups	*p*-Value
		Baseline Versus End of Treatment	Baseline Versus End of Treatment		(Intervention vs. Controls)
Jenks et al. 2010 [26]	BASFI10 response	3.5 (2.0) vs. 2.9 (1.9)	3.6 (1.9) vs. 3.1 (2.2)	−0.1 (−0.7 to −0.6)	$p = 0.839$
	BASDAI	4.2 (2.2) vs. 3.2 (2.1)	4.5 (2.0) vs. 3.9 (2.2)	−0.6 (−1.6 to −0.3)	$p = 0.182$
	ASAS20 response	6/32 (18.8%) [a]	9/31 (25.8%) [a]		$p = 0.338$
	VAS pain	2.9 (2.3) vs. 2.7 (2.5)	3.0 (2.6) vs. 2.6 (2.2)	0.2 (−0.8 to −1.1)	NR
	VAS fatigue	3.4 (2.2) vs. 2.7 (2.0)	4.0 (2.4) vs. 3.3 (2.4)	−0.4 (−1.4 to −0.7)	NR
	ASQoL	6.9 (4.5) vs. 4.9 (4.8)	6.4 (4.3) vs. 5 (4.7)	−0.5 (−2.0 to −1.1)	NR
	SJC	0.4 (0.9) vs. 0.25 (0.9)	0.5 (1.1) vs. 0.2 (0.5)	0.1 (−0.2 to −0.4)	NR
	TJC	2.0 (2.1) vs. 3.1 (3.9)	2.6 (2.6) vs. 5.4 (8.8)	−1.6 (−4.6 to −1.5)	NR
	CRP	6.8 (6.7) vs. 6.7 (6.3)	10.0 (11.3) vs. 11.3 (11.2)	−3.5 (−7.8 to −0.8)	NR
Brophy et al. 2008 [27]	VAS activity	4.1 (2.2) vs. 3.6 (2.6)	3.5 (1.9) vs. 2.9 (2.2)	0.20 (−0.47 to 0.86)	NR
	Global well-being	4.1 (2.5) vs. 3.7 (3.0)	3.2 (2.0) vs. 2.9 (2.3)	0.16 (−0.61 to 0.93)	NR
	Bowel symptoms	Diarrhea:			
		1.8 (2.6) vs. 1.4 (2.3)	1.9 (2.7) vs. 1.0 (1.7)	0.24 (−0.36 to 0.83)	NR
		Stomach pain:			
		2.0 (2.7) vs. 1.4 (2.2)	2.1 (2.6) vs. 1.2 (1.6)	0.17 (−0.42 to 0.76)	NR
		Blood in stools:			
		0.6 (1.6) vs. 0.4 (1.1)	0.6 (1.8) vs. 0.5 (1.6)	−0.14 (−0.55 to 0.27)	NR

Data are presented as the mean (standard deviation) except contrary mention. [a] Data presented as number of patients (percentages). ASAS: Assessment of Spondyloarthritis International Society; ASQoL: Ankylosing Spondylitis Quality of Life; BASDAI: Bath Ankylosing Spondylitis Disease Activity Index; BASFI: Bath Ankylosing Spondylitis Functional Index; CRP: C-reactive Protein; NR: Not Reported; SJC: Swollen Joint Count; TJC: Tender Joint Count; VAS: Visual Analogic Scale.
